# Supplementary material for: MDM2-Dependent Rewiring of Metabolomic and Lipidomic Profiles in Dedifferentiated Liposarcoma Models
Source: Cancers (Basel). 2020 Aug 4;12(8):2157. doi: 10.3390/cancers12082157 (PMC7463633; doi:10.3390/cancers12082157)
Supplement: Supplementary file 1 [file cancers-12-02157-s001.zip › Supplementary_file_1.pdf]

		Metabolome		Lipidome			
**Cell Line**	**MDM2 Status**	**DMEM**	**DMEM + RG7112**	**DMEM**	**STDP**	**DMEM + AT**	**STDP + AT**
**LPS141**	High	3	3	3	3	3	3
**Lipo-246**	High	5	3	3	3	3	3
**Lipo-224A**	High	5	0	3	3	3	3
**224B**	Low	3	0	3	3	3	3
**815**	Low	5	3	3	3	3	3
**863**	Low	5	3	3	3	3	3

Supplementary Table 1: Sarcoma cell line characteristics for metabolomic and lipidomic profiling.
DMEM - Dulbecco's Modified Eagle's media supplemented with fetal bovine serum
RG7112 – MDM2/P53 binding inhibitor
STDP – sterol-deprived media
AT - atorvastatin

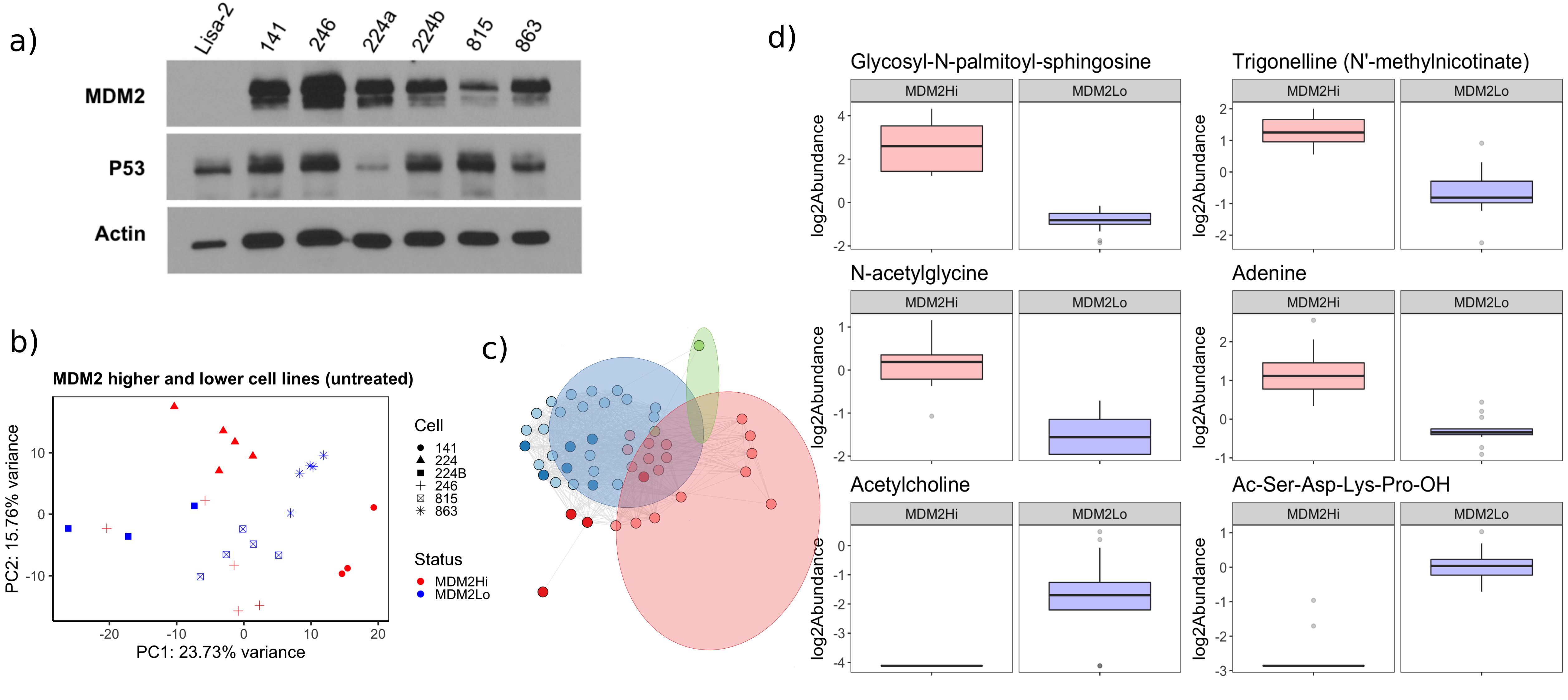

Figure S1: MDM2 status and metabolomic (Metabolon) profiling in DDLPS cell lines.
a) MDM2 protein levels in DDLPS cell lines. b) Principal Components Analysis of cell lines with metabolomic (Metabolon) profiling, showing clear clustering of replicate samples. c) Visual of the final network model used for enrichment. Colors correspond to modules detected by Louvain clustering. Darker nodes (e.g. dark red) are “seed” nodes found different in MDM2 high and low in our panel, while lighter nodes (e.g. light red) were recruited from our RaMP database (see methods). d) Boxplot distribution of abundance levels for metabolites of interest that are altered by MDM2 status (FDR-adjusted p-val < 0.05 and |log2 FC| > 0.75).

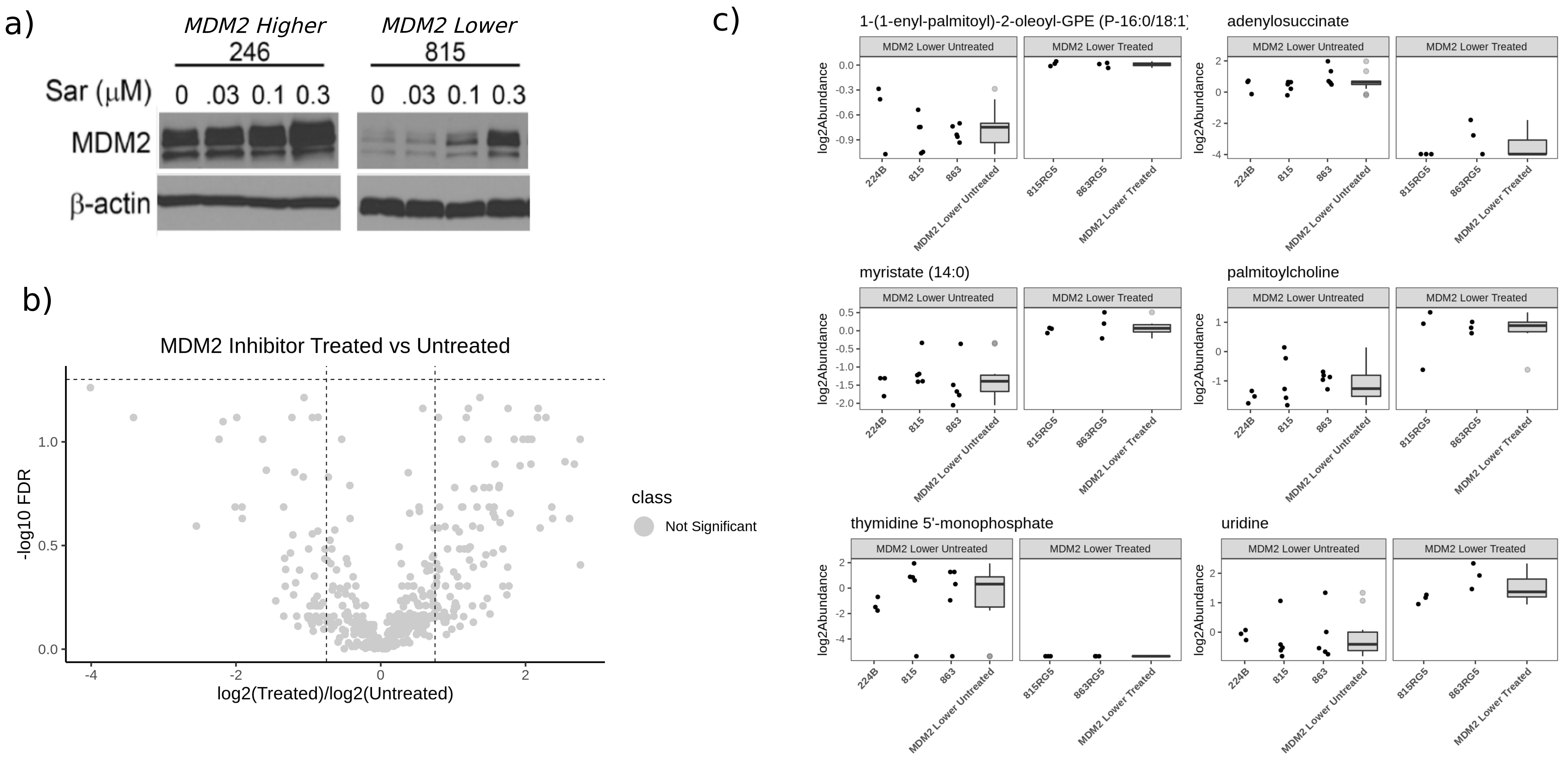

Figure S2: Comparing the effect of MDM2 inhibitor in DDLPS cells.
a) MDM2 protein levels in MDM2 higher amplification (246) and MDM2 lower amplification (815) cells treated with MDM2 inhibitor. b) Comparison of metabolite levels between MDM2 higher and lower cells after MDM2 inhibitor treatment. c) Metabolites altered by MDM2 inhibitor treatment in DDLPS cells with lower MDM2 copy number.

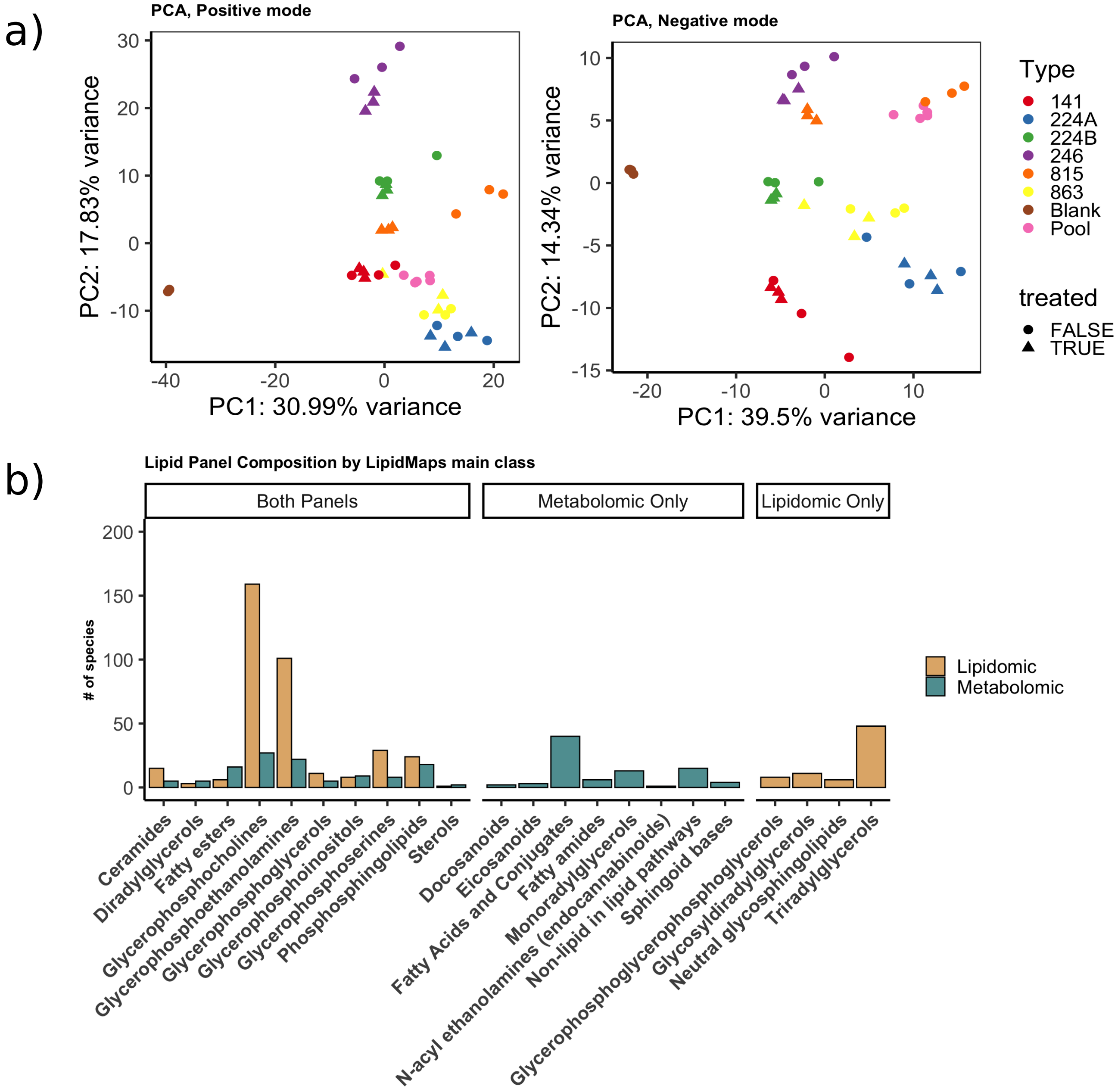

c)

Lipid ID	Metab pval	Metab log2fc	Lipid pval	Lipid log2fc
GlcCer_NS(d18:1/16:0)	0.03	3.63	0.02	2.39
PC(18:2_18:2)	0.33	1.48	0.90	0.86
Cer-NS(d18:1/16:0)	0.15	1.45	0.80	1.45
PE(18:1_18:2)	0.06	1.40	0.71	1.33
PE(18:0_20:4)	0.01	1.29	0.82	0.73
SM(d18:1/24:1)	0.13	-0.78	0.02	-1.27
1-oleoyl-GPC (18:1)	0.49	-0.80	0.85	-0.63
SM(d18:2/24:1)	0.37	-0.85	0.10	-1.20
LPC(16:1)	0.56	-1.27	0.91	-1.03
SM(d17:1/24:1)	0.14	-1.46	0.31	-14.81

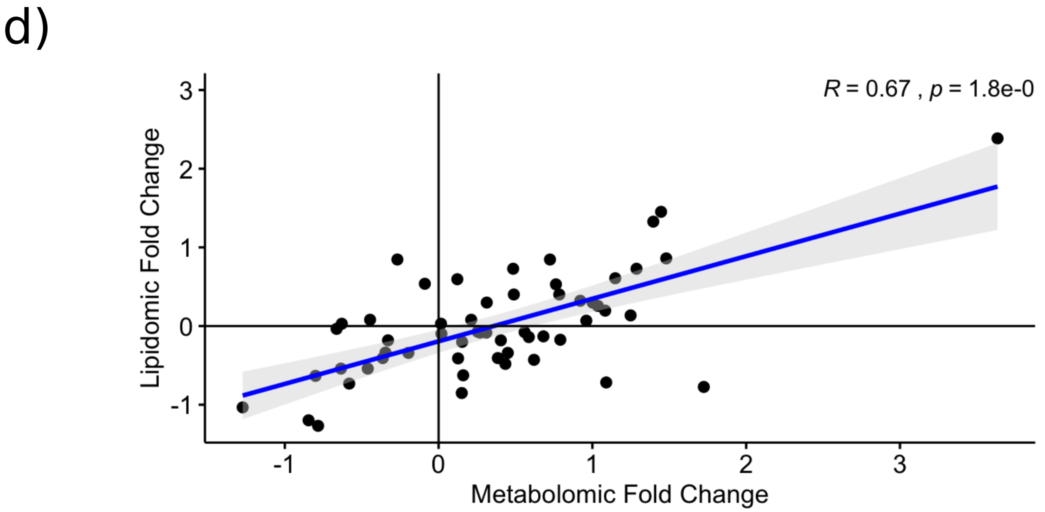

Figure S3: Quality assessment of lipidomic data.
a) Principal Components Analysis shows clustering of quality control samples, and clustering by MDM2 status and treatment status in cells. b) Overlap in coverage between the Metabolon and lipidomic platforms. c) Concordance of alterations in key lipids between metabolomic and lipidomic platforms. d) Correlation of fold change between MDM2 higher and lower cell lines in lipid species found in both panels.

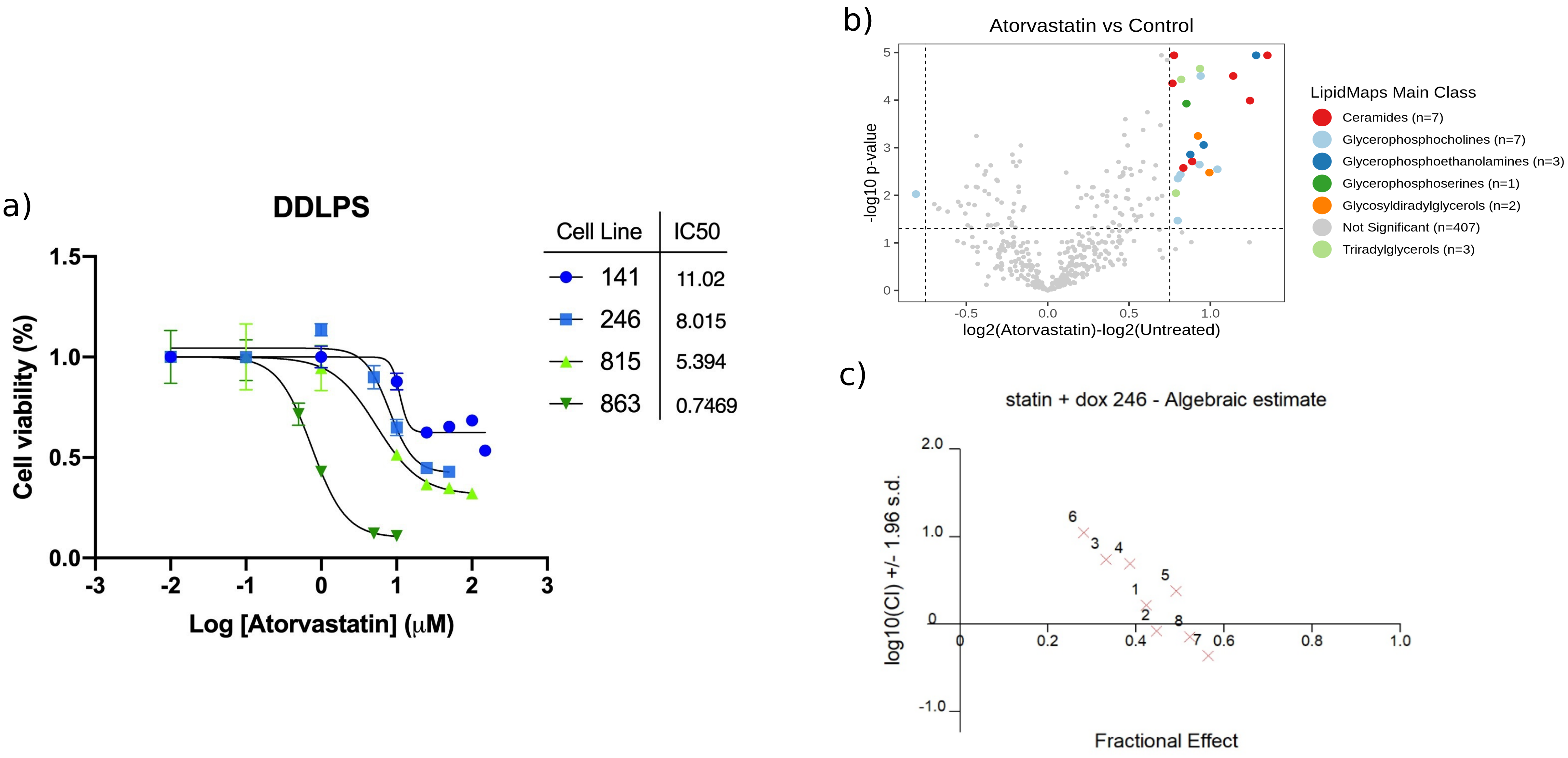

Figure S4: Effect of MDM2 amplification/Atorvastatin treatment in DDLPS cells.
a) MDM2 lower amplification cells (815, 863) are more sensitive to statin treatment than MDM2 higher amplification cells (141, 246). b) Statin treatment causes a global up-regulation of lipids, including ceramides, in all DDLPS cells grown in control media, independent of MDM2 status. c) Multiple concentrations of atorvastatin and doxorubicin were evaluated for effect on cell viability. Overall, the use of atorvastatin along with doxorubicin demonstrated clear antagonism (points above the x-axis). Points below the the x-axis demonstrate synergy.
